# Mechanical Properties of Granite Residual Soil Reinforced by Permeable Water-Reactive Polyurethane

**DOI:** 10.3390/polym18030381

**Published:** 2026-01-30

**Authors:** Shuzhong Tan, Jinyong Li, Dingfeng Cao, Tao Xiao, Jiajia Zheng

**Affiliations:** 1 Fujian Provincial Center for Transportation Construction Quality and Safety, Fuzhou 350001, China; 2 Nanping Wusha Expressway Co., Ltd., Jianyang, Nanping 354200, China; 3 A State Key Laboratory for Tunnel Engineering, School of Civil Engineering, Sun Yat-sen University, Zhuhai 519082, China

**Keywords:** granite residual soil (GRS), permeable water-reactive polyurethane (PWPU), acoustic emission (AE), digital image correlation (DIC), uniaxial compressive strength (UCS), micromechanism

## Abstract

Granite residual soil (GRS) is highly susceptible to water-induced softening, posing significant risks of slope instability and collapse. Conventional impermeable grouting often exacerbates these hazards by blocking groundwater drainage. This study investigates the efficacy of a permeable water-reactive polyurethane (PWPU) in stabilizing GRS, aiming to resolve the conflict between mechanical reinforcement and hydraulic conductivity. Uniaxial compression tests were conducted on specimens with varying initial water contents (5%, 10%, and 15%) and PWPU contents (5%, 10%, and 15%). To reveal the multi-scale failure mechanism, synchronous acoustic emission (AE) monitoring and digital image correlation (DIC) were employed, complemented by scanning electron microscopy (SEM) for microstructural characterization. Results indicate that PWPU treatment significantly enhances soil ductility, shifting the failure mode from brittle fracturing to strain-hardening, particularly at higher moisture levels where failure strains exceeded 30%. This enhancement is attributed to the formation of a flexible polymer network that acts as a micro-reinforcement system to restrict particle sliding and dissipate strain energy. An optimal PWPU content of 10% yielded a maximum compressive strength of 4.5 MPa, while failure strain increased linearly with polymer dosage. SEM analysis confirmed the formation of a porous, reticulated polymer network that effectively bonds soil particles while preserving permeability. The synchronous monitoring quantitatively bridged the gap between internal micro-crack evolution and macroscopic strain localization, with AE analysis revealing that tensile cracking accounted for 79.17% to 96.35% of the total failure events.

## 1. Introduction

Granite residual soil (GRS), originating from the intense in situ physical and chemical weathering of granite parent rock under hot and humid climatic conditions, is extensively distributed in tropical and subtropical regions, notably South China [1,2]. Fundamentally characterized by a loose structure and a high void ratio, the physical mechanical properties of GRS vary significantly depending on the degree of weathering [3]. Despite its initial strength, GRS possesses a critical vulnerability regarding its high sensitivity to water. Upon moisture infiltration, the soil structure tends to soften and disintegrate rapidly, leading to a sharp reduction in shear strength under stress [4,5]. Consequently, engineering projects in GRS strata frequently encounter severe geohazards, including surface collapse, instability of deep excavations, and slope failures [6,7].

Conventional soil stabilization techniques, encompassing inorganic binders like cement and organic polymers like epoxy resins, are extensively utilized to enhance the mechanical properties of problematic soils [8]. However, these established methods exhibit inherent drawbacks regarding their solidification characteristics. Cement-treated soils often exhibit high brittleness, leading to sudden and catastrophic failure without warning signals when subjected to dynamic loads [7,9]. Crucially for underground engineering, traditional cement grouting tends to fill soil pores completely, forming an impermeable barrier that blocks natural groundwater drainage pathways. This blockage leads to the dangerous accumulation of pore water pressure behind the reinforced zone and potentially induces lining cracking or slope instability. Similarly, organic binders like epoxy resins, despite their renowned adhesive strength, are often limited by high viscosity [10]. This characteristic restricts their penetrability into the fine micro-pores of clay-rich soil matrices. Furthermore, the cured epoxy matrix is typically rigid and brittle, resulting in poor compatibility with the deformation of the surrounding soil.

In response to these limitations, polymer grouting techniques, particularly using polyurethane (PU), have emerged as a promising alternative. Unlike traditional rigid binders, PU materials are characterized by rapid reaction kinetics, excellent environmental durability, and adjustable mechanical properties ranging from rigid to flexible [11,12]. However, standard PU grouts are typically hydrophobic, designed to expand aggressively to seal fissures and cut off groundwater pathways. While effective for waterproofing, this sealing effect mirrors the drawbacks of cement by blocking drainage, which is detrimental to the stability of water-sensitive slopes. To overcome this bottleneck, permeable water-reactive polyurethane (PWPU) has been developed [13,14]. Specifically engineered to resolve the conflict between reinforcement and drainage, PWPU reacts with pore water to form a solidified network that bonds soil particles effectively while preserving a porous structure. This unique mechanism allows for the continuous dissipation of excess pore water pressure, making it an ideal candidate for stabilizing soil strata where both mechanical enhancement and hydraulic conductivity are critical [15,16]. Nevertheless, existing research has not fully addressed the application of this material to GRS. Although the mechanical behavior of unsaturated soils has been widely investigated within the framework of matric suction [17], the stabilization mechanism of PWPU introduces a unique chemo-mechanical coupling that remains underexplored. Unlike traditional chemical grouting, where excessive pore water typically dilutes the binder and compromises strength, in PWPU-treated systems, the pore water serves as a critical reactant governing the polymer’s cellular expansion and final microstructure. Existing research has not fully elucidated how the initial unsaturation level regulates the competition between ‘water-induced softening’ of the soil aggregates and ‘water-enhanced foaming’ of the polymer matrix. Consequently, the specific failure evolution and brittle-to-ductile transition of PWPU-reinforced GRS under varying unsaturated conditions require systematic investigation.

To thoroughly reveal the underlying interaction mechanism between the polymer network and the soil matrix, advanced non-destructive monitoring techniques are essential. Among these, acoustic emission (AE) technology is widely recognized as an effective tool for tracking internal damage evolution. This method detects transient elastic waves generated by the rapid release of energy from localized sources, allowing for the real-time monitoring of micro-crack initiation and propagation. Current research has extensively utilized AE parameters to characterize the fracture process in brittle materials, including concrete and cemented sands [16,18]. However, regarding polymer-reinforced soils, the application of AE remains challenging due to the viscoelastic nature of the polymer network. The distinct signal attenuation characteristics and the specific acoustic signatures associated with the debonding of the flexible polymer-soil interface have not been fully deciphered. Complementing this internal monitoring, digital image correlation (DIC) serves as a powerful optical technique for measuring surface deformation. Unlike traditional point-based strain gauges, DIC offers full-field strain measurement and visualization, accurately capturing the development of shear bands and strain localization zones during loading. While DIC has been successfully employed to visualize failure modes in various geotechnical materials, relying solely on surface deformation fails to provide a complete picture of the internal damage accumulation that precipitates macroscopic failure. Furthermore, the synchronous application of AE and DIC to investigate the coupling effect of polymer ductility and soil granularity in GRS remains unexplored. Establishing a direct quantitative correlation between the internal micro-fracturing detected by AE and the external strain localization observed by DIC is critical. This multi-scale approach is necessary to bridge the gap in understanding the progressive failure mechanism of PWPU-treated GRS (PWPU-GRS).

In this study, a novel permeable PWPU was employed to reinforce granite residual soil. A series of unconfined compressive strength (UCS) tests were conducted on GRS specimens with varying polymer contents. The mechanical properties, including strength, stiffness, and ductility, were evaluated. Simultaneous monitoring using AE and DIC techniques was performed to reveal the real-time damage evolution and strain localization characteristics. Furthermore, scanning electron microscopy (SEM) was utilized to uncover the micro-bonding mechanism between the PWPU matrix and soil particles. Unlike conventional impermeable grouting, the PWPU material is chemically engineered to generate an interconnected porous structure, which naturally facilitates drainage. Therefore, the primary challenge addressed in this study is to determine whether this porous composite can maintain sufficient mechanical stability to reinforce the soil skeleton without collapsing. This research aims to rigorously establish the mechanical viability and failure mechanisms of the composite, providing the structural prerequisite for its subsequent hydraulic applications.

## 2. Materials and Methods

### 2.1. Materials

#### 2.1.1. Granite Residual Soil

**Figure 1:**
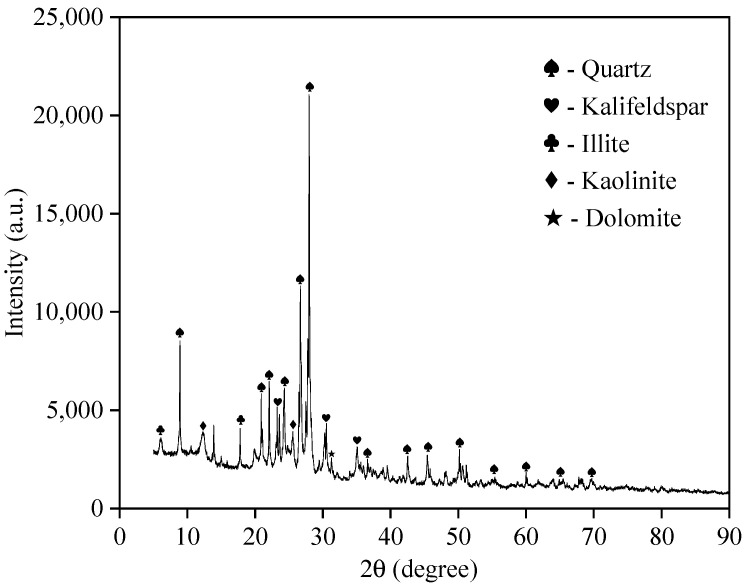
Mineral composition of the test soil measured by X-ray diffraction analysis (XRD).

**Figure 2:**
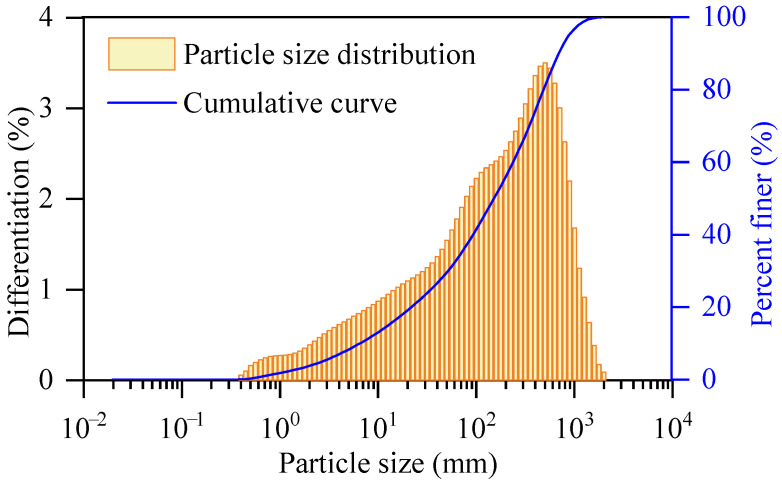
Soil particle distribution measured by laser particle size analyzer.

The GRS utilized in this study was collected from a construction excavation site in Nanping, Fujian Province, China. The soil exhibits a distinctive reddish-brown color, which is characteristic of the extensive chemical weathering of parent rocks in tropical and subtropical marine environments. Prior to the experiments, the natural soil was air-dried for two weeks, pulverized, and passed through a 2 mm sieve to remove coarse gravel and organic debris, ensuring the homogeneity of the specimens [16]. To characterize the material properties, the mineral composition was analyzed using X-ray diffraction (XRD), as presented in Figure 1. The chemical oxide components were quantitatively measured by an X-ray Fluorescence Spectrometer (XRF), and the results are listed in Table 1. Additionally, the particle size distribution of the tested soil is illustrated in Figure 2. As shown in the figure, the curve plots both the differentiation percentage and the cumulative passing percentage against particle size, revealing the gradation characteristics of the soil matrix used in this study.

#### 2.1.2. Permeable Water-Reactive Polyurethane

**Figure 3:**
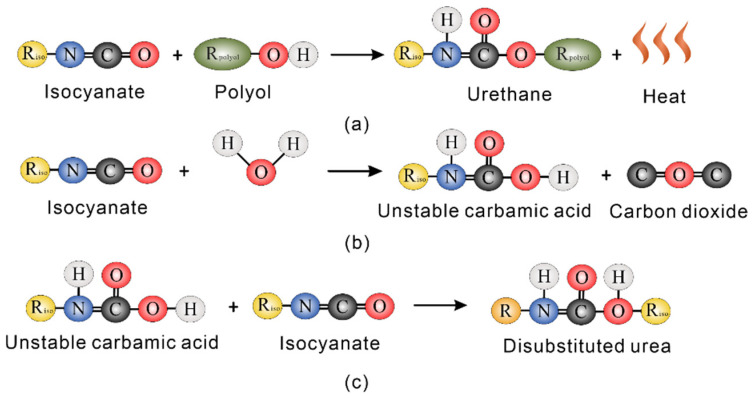
Schematic diagram of the reaction between polyurethane and water, (**a**) urethane production, (**b**) reaction of the isocyanate with water, and (**c**) disubstituted urea production.

The stabilizing agent employed is a two-component hydrophilic PWPU, supplied by Wanhua Chemical (Ningbo, China) Rongwei Polyurethane Co., Ltd. The system consists of Component A (primarily isocyanate prepolymers) and Component B (a blend of polyols, surfactants, and catalysts). The material is characterized by a bulk density of 1.05 ± 0.05 g/cm^3^. The solidification process is driven by complex chemical reactions initiated upon mixing the components with pore water, as visually summarized in Figure 3 [19]. As depicted in Figure 3a, the fundamental polymerization occurs between the isocyanate groups (-NCO) and polyols to form urethane linkages, releasing heat during the exothermic reaction. Simultaneously, the isocyanate groups react with water to produce unstable carbamic acid, which subsequently decomposes into amines and CO_2_ gas, as shown in Figure 3b. This generation of CO_2_ induces volume expansion, creating a porous reticulated structure that ensures permeability. Furthermore, as the reaction proceeds, the generated amines react with the remaining isocyanate groups to form stable disubstituted urea linkages (Figure 3c). The resulting polymer matrix acts as an effective bonding agent that coats soil particles and enhances the hydro-mechanical stability of the GRS.

### 2.2. Laboratory Test Procedure

**Figure 4:**
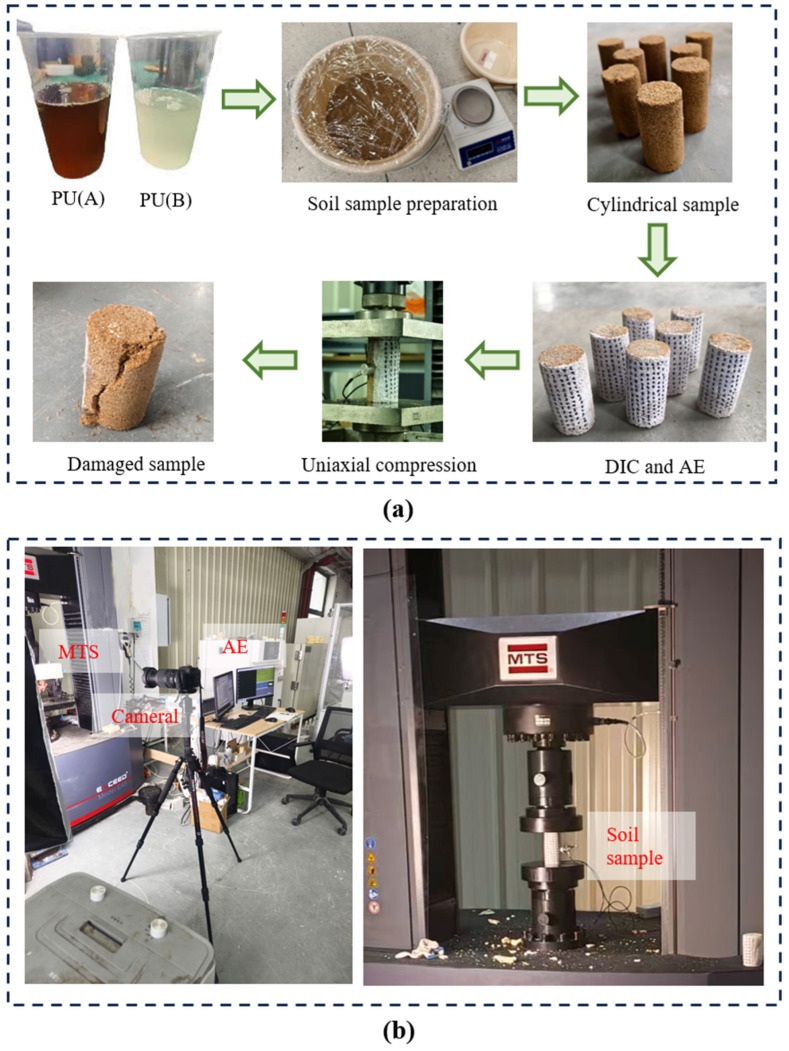
Configuration of the test, (**a**) samples preparation process, and (**b**) loading equipment.

The experimental program was designed to systematically investigate the influence of initial water content and PWPU content on the mechanical performance of the reinforced soil. Test variables included initial water contents of 5%, 10%, and 15% and PWPU contents of 5%, 10%, and 15%, while the dry density was maintained 1.55 g/cm^3^ across all groups. Specimen preparation followed a strict mixing and compaction protocol. Air-dried GRS was mixed with distilled water to reach the target moisture levels and sealed in plastic bags for 24 h to ensure equilibrium in accordance with the Standard for Geotechnical Testing Method (GB/T 50123-2019). The predetermined mass of PWPU was added to the wet soil and agitated quickly for 30 s using a mechanical mixer to ensure homogeneous distribution. The mixture was compacted into a cylindrical mold in layers with a thickness of 25 mm. To ensure structural integrity, the surface of each lower layer was scarified prior to adding the subsequent layer. A thin layer of petroleum jelly was applied to the inner wall of the mold to facilitate demolding. Due to the rapid reaction kinetics of the PWPU material, the primary solidification process is essentially completed within a short timeframe. Therefore, after finishing the final layer, specimens were allowed to cure before being demolded and stored in a dry, shaded area for 24 h to ensure full strength development and structural equilibrium prior to testing, as shown in Figure 4a.

UCS tests were conducted using a computer-controlled MTS-E45.305 material testing system as shown in Figure 4b. This system synchronously recorded time, axial displacement, and reaction force during the testing process. The actuator featured a displacement resolution of 1.7 × 10^−5^ mm. Tests were executed under displacement control with a constant loading rate. During the loading process, synchronous AE monitoring was performed using a DS5-16C system (Beijing Soft Island Science and Technology Co., Ltd., Beijing, China). To filter out environmental background noise, the preamplifier gain was set to 40 dB with an acquisition threshold of 10 mV. AE signals were captured using two broadband piezoelectric sensors featuring a frequency response range of 50 kHz to 800 kHz, with a sampling rate set to 3 MHz. To optimize signal collection, sensors were symmetrically mounted on the central section of the specimen, identified as the zone prone to crack initiation. A specialized silicone grease couplant was applied between the sensors and the specimen surface to eliminate air gaps and minimize signal attenuation. Prior to formal testing, Hsu-Nielsen source tests (pencil lead break tests) were performed on the specimen surface to calibrate the threshold and verify the signal reception sensitivity of each channel. The AE acquisition system was time-synchronized with the MTS loading frame to ensure data correspondence. DIC was employed as a non-contact optical measurement technique to capture the full-field deformation response of the specimens. Based on principles of digital image processing and numerical computation, this method determines displacement and strain distributions by tracking the movement of surface features between reference (undeformed) and target (deformed) images via correlation algorithms. In this study, a high-definition digital camera (Canon, Zhuhai, China) was utilized to record the surface evolution during testing, and the open-source software Ncorr was subsequently used to compute the strain fields. The DIC algorithm used in this research has been elaborated by Blaber et al. [20]. To optimize the correlation quality and measurement accuracy, an artificial random speckle pattern was fabricated on the specimen surface prior to loading to provide high-contrast tracking markers.

Microscopic interaction mechanisms were investigated using an Axia ChemiSEM from Thermo Fisher Scientific (Waltham, MA, USA). Prior to SEM analysis, cubic samples (10 × 10 × 10 mm) were extracted from the central region of the specimens and subjected to dehydration and drying according to established protocols. Due to the non-conductive nature of the soil-polymer matrix, gold sputter coating was performed to prevent charge accumulation. The coating parameters were strictly controlled to balance conductivity with the preservation of surface details, as excessive coating thickness can obscure the original microstructure. Consequently, a coating current of 20 mA, a sputtering speed of 10 mm/min, and a duration of 120 s were selected to ensure high-quality imaging without structural masking.

## 3. Results and Discussion

### 3.1. Strain-Stress Relationship

**Figure 5:**
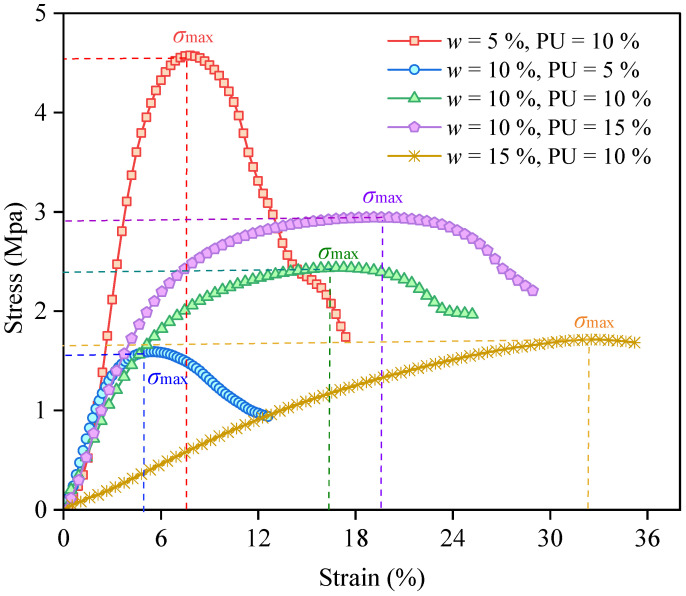
Stress–strain curves of PWPU-reinforced GRS with varying initial water contents (*w*) and PWPU contents (PU).

The typical stress–strain curves of the PWPU-reinforced GRS under uniaxial compression with varying initial water contents (*w*) and PWPU contents (PU) are presented in Figure 5. The mechanical response exhibits distinct phases, namely, an initial linear elastic stage, a yielding stage, and a post-peak failure stage. The morphology of these curves is significantly governed by both the hydration state of the soil and the dosage of the polymer binder [21]. The addition of PWPU significantly enhances the ductility of the soil. Taking the *w* = 10% group as an example, increasing the PU content from 5% to 15% leads to a substantial increase in the failure strain. The specimen with 15% PU content shows a much more extended yielding plateau compared to the 5% PU specimen, indicating that a higher density of polymer cross-links effectively bridges soil particles and restrains crack propagation [22,23]. The influence of water content on the failure mode is profound. As observed in Figure 5, at a constant PU content of 10%, the specimen with low initial water content (*w* = 5%) exhibits a distinct brittle failure behavior. The stress increases sharply to a peak value of approximately 4.5 MPa and then drops abruptly, indicating a rapid loss of bearing capacity due to the fracture of the rigid soil-polymer skeleton [24]. With the increase in initial water content to *w* = 10%, the curve becomes broader, and the post-peak softening is less severe, demonstrating a transition towards ductile behavior [25]. Notably, when the water content reaches *w* = 15%, the material exhibits a strain-hardening characteristic. The stress continues to rise or stabilize even at large axial strains (>30%) without a distinct peak drop. This phenomenon suggests that higher water content facilitates the formation of a more flexible, foam-like polymer network due to increased CO_2_ generation and expansion, which allows for significant deformation while maintaining residual integrity [26]. This behavior is distinguished from geometric confinement effects by the DIC observations, which show a uniform strain distribution across the central region rather than localized end constraints. Furthermore, the dominance of tensile cracking detected by AE confirms that the hardening stems from the continuous stretching of the polymer ligaments.

**Figure 6:**
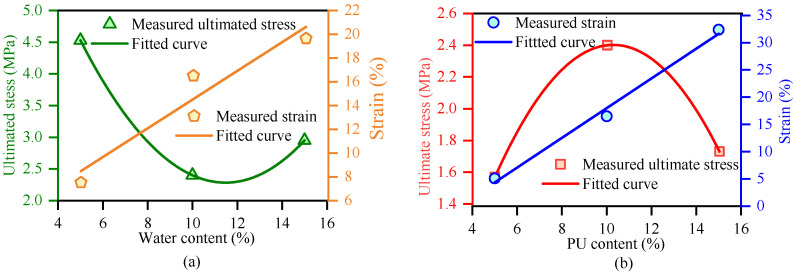
Variations in ultimate stress and failure strain with (**a**) initial water content and (**b**) PWPU content.

To further quantify these effects, the variations in ultimate stress and failure strain are summarized in Figure 6. Figure 6a depicts the influence of initial water content on the mechanical parameters. Consistent with the stress–strain curves, the failure strain (orange line) increases significantly with water content, reflecting the transition from a rigid to a flexible matrix. The ultimate stress (green curve), however, exhibits a distinctive “U-shaped” trend. It initially decreases as water content increases from 5% to 10%, a phenomenon typical for unsaturated soils attributed to the sharp reduction in matric suction and the softening of soil aggregates. However, a slight yet critical recovery in strength is observed at higher water contents (*w* = 15%). This recovery suggests a competitive mechanism: while excess water weakens the soil skeleton, it simultaneously acts as a reactant that ensures the fuller hydration of the PWPU. The resulting highly developed reticulated polymer network compensates for the loss of inter-particle friction, thereby restoring structural integrity. A similar interaction mechanism was reported by Vydehi and Moghal (2022) [27], where the biopolymer strands were observed to encapsulate soil particles and form cohesive bridges across void spaces.

Regarding the influence of polymer dosage, Figure 6b illustrates a different pattern. The failure strain (blue line) exhibits a robust positive linear correlation with polymer dosage, confirming that the deformation capacity is strictly controlled by the volume fraction of the flexible polymer phase. In contrast, the ultimate stress (red curve) displays a non-monotonic parabolic trend, reaching a peak value at an optimal PU content of 10%. The unexpected decline in strength at the highest dosage (PU = 15%) warrants close attention and can be attributed to two primary factors. First, an excessive volume of polymer paste creates a “lubrication effect,” separating the soil particles and preventing direct grain-to-grain contact, thus reducing the frictional component of shear strength. Second, the intense foaming reaction at high polymer concentrations may generate excessive macro-voids, which act as stress concentrators within the specimen. Consequently, a PWPU content of 10% is identified as the optimal threshold, effectively balancing the “bridging strengthening” against the adverse “lubrication weakening” to achieve maximum bearing capacity.

### 3.2. Microcrack Formation Detection Using AE

**Figure 7:**
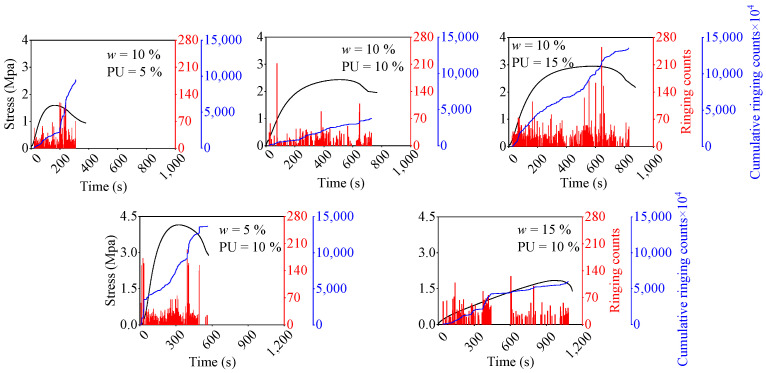
Relationship between AE ringing counts (in red), cumulative AE counts (blue curve), and load path (black curve) under different contents of water and PU contents.

To elucidate the internal damage evolution characteristics of the PWPU-reinforced GRS, the temporal evolution of AE ringing counts and cumulative counts superimposed on the stress history is presented in Figure 7. The damage progression exhibits distinct patterns contingent upon the hydration state and polymer dosage.

For the specimen with low water content (*w* = 5%, PU = 10%), the AE activity remains relatively quiescent during the initial elastic and pre-peak stages, as shown in the bottom-left of Figure 7. A sudden, explosive surge in ringing counts is observed only near the peak stress, coinciding with an abrupt step-like jump in the cumulative count curve. This phenomenon indicates a brittle failure mode, where elastic energy accumulates without significant dissipation until a critical threshold is reached, leading to the instantaneous fracture of the rigid soil-polymer skeleton. In contrast, specimens with higher water contents (*w* = 15%) or higher polymer contents (PU = 15%) display a more continuous and progressive acoustic signature. As illustrated in the right-side subplots of Figure 7, the ringing counts are densely distributed throughout the entire loading process, including the post-peak softening stage. The cumulative ringing count curve rises smoothly and gradually. This behavior suggests that the flexible polymer network facilitates a progressive failure mechanism. The hydration reaction with higher water content produces a more porous and ductile foam structure, which undergoes gradual stretching and localized debonding rather than catastrophic breakage, thereby enhancing the energy absorption capacity of the composite [16,28].

**Figure 8:**
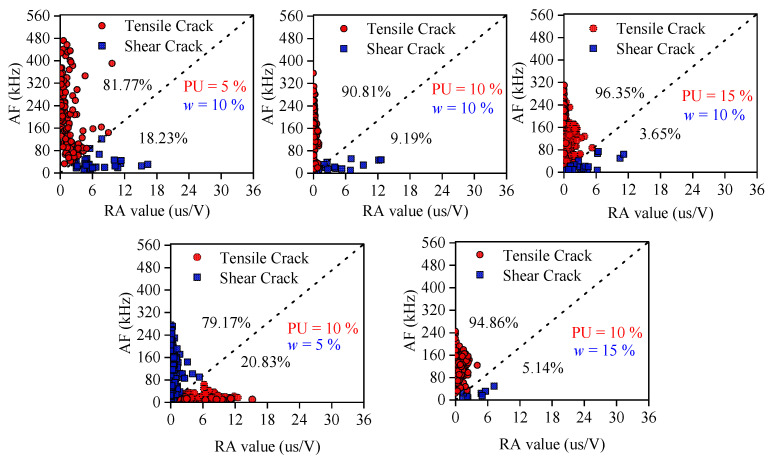
Relationship between average frequency (AF) and RA values.

To classify the microscopic failure mechanisms and decipher the wave mode characteristics of the internal damage, the AE signals were quantitatively analyzed utilizing the RA value (Rise Time/Amplitude) and AF (Average Frequency) method, as depicted in Figure 8 [29,30]. The dashed diagonal line represents the threshold separating tensile cracks (lower right) from shear cracks (upper left). In the classification framework, the waveform parameters serve as a fingerprint for the fracture mode, where shear cracks typically generate transverse waves characterized by slower propagation, longer rise times, and lower frequencies (high RA, low AF), whereas tensile cracks produce longitudinal waves with rapid rise times and higher frequency components (low RA, high AF) [31]. The statistical distribution relative to the diagonal threshold line reveals that tensile cracking is the predominant failure mode across all experimental groups, accounting for over 79% of the total acoustic events. This fundamental observation indicates that the cohesive strength provided by the polymer binder fundamentally alters the failure kinematics of the granular soil. Unlike untreated soil, where failure is governed by inter-particle frictional sliding, the reinforced matrix fails primarily through the rupture of the bonding agents.

Crucially, the evolution of crack types is highly sensitive to the material composition. As the PWPU content increases from 5% to 15% (at *w* = 10%), the proportion of tensile cracks rises significantly from 81.77% to 96.35%. This trend suggests that with a higher dosage of binder, the soil particles are more deeply embedded within a continuous polymer network. Consequently, the failure process shifts from the debonding of weak interfaces to the tearing of the bulk polymer matrix itself [32].

Similarly, increasing the initial water content from 5% to 15% (at PU = 10%) elevates the tensile crack ratio from 79.17% to 94.86%. This can be attributed to the morphological change in the polymer matrix; higher water content promotes foaming, creating a cellular structure composed of numerous thin, flexible pore walls. Under compressive loading, these micro-walls undergo extensive stretching and bending, inherently tensile deformation modes rather than shearing. This dominance of tensile micro-cracking provides the physical explanation for the macroscopic ductility and strain-hardening behavior observed in the mechanical tests. The bridge effect of the PWPU network effectively suppresses the relative sliding between soil grains. By constraining particle rotation and displacement, the polymer network converts the macroscopic compressive and shear stresses into microscopic tensile stresses within the flexible polymer ligaments. This conversion mechanism allows the material to dissipate substantial energy through the elongation of the polymer chains, preventing the formation of catastrophic shear planes and ensuring the structural integrity of the reinforced soil even at large deformations [33].

### 3.3. Strain and Displacement Distribution and Macroscopic Cracks Production Analysis

**Figure 9:**
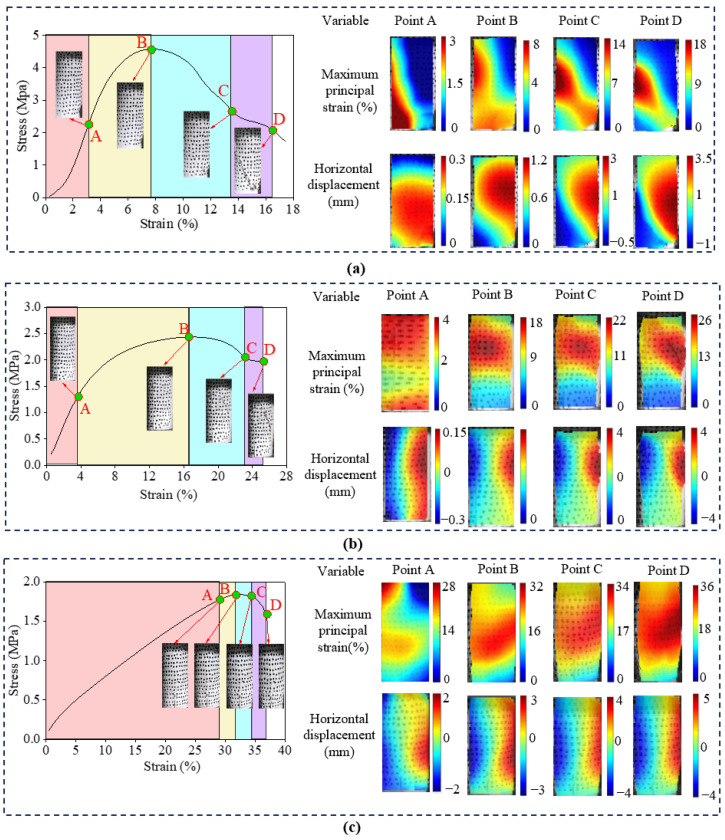
Digital images correlation (DIC) results under conditions of different initial water contents (*w*): (**a**) *w* = 5%, PU = 10%; (**b**) *w* = 10%, PU = 10% and (**c**) *w* = 15%, PU = 10%.

To visually characterize the multi-scale failure mechanisms, the full-field maximum principal strain and horizontal displacement contours were analyzed utilizing the DIC technique [34]. The influence of initial water content on the macroscopic failure pattern is illustrated in the series of Figure 9. As presented in Figure 9a, the specimen with low water content exhibits pronounced strain localization. At the peak stress stage (Point B), a narrow, high-intensity diagonal shear band forms rapidly traversing the specimen. The horizontal displacement field reveals a sharp discontinuity across this band, indicating significant relative sliding between the soil blocks. This observation signifies a brittle shear failure mode driven by the fracture of the rigid soil-polymer skeleton.

With an increase in water content to 10% (Figure 9b), the shear band widens significantly. While the diagonal orientation persists, the strain concentration gradients are less severe compared to the 5% group, reflecting a transition towards a more distributed deformation mode. At high water content (Figure 9c), the failure mode shifts completely to ductile barreling. No singular macroscopic shear plane is observed. Instead, the maximum principal strain is distributed broadly across the central region, and the horizontal displacement field is symmetrical. This drum-like expansion confirms that the abundant gas-filled pores generated by the water-isocyanate reaction allow the material to accommodate large deformations without disintegration [35].

**Figure 10:**
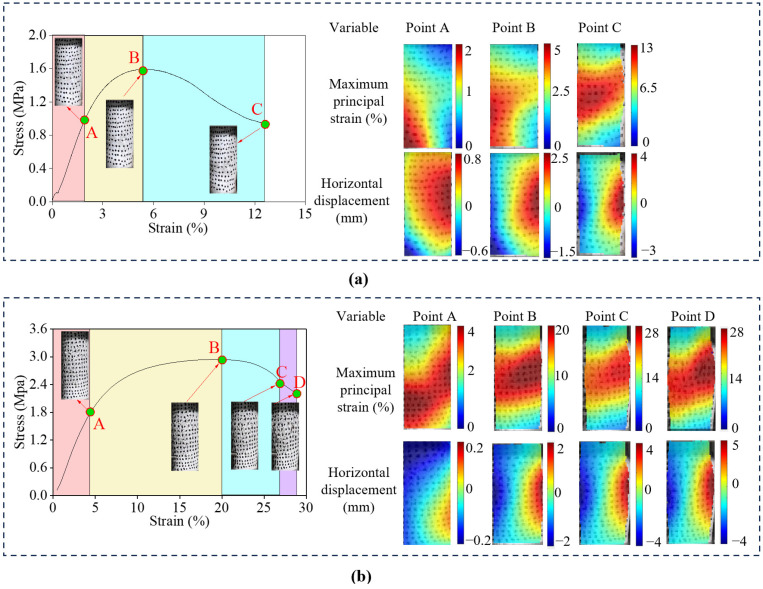
Digital images correlation (DIC) results under conditions of different PWPU contents (PU): (**a**) *w* = 10%, PU = 5%; (**b**) *w* = 10%, PU = 15%.

By integrating Figure 9b with Figure 10, the progressive effect of PWPU content can be systematically analyzed. As presented in Figure 10a, the specimen with a low polymer dosage exhibits a relatively weak and non-uniform response. Strain localization emerges early at the peak stress stage (Point B), and the ultimate failure is characterized by a localized shear zone, leading to a significantly lower peak stress. With the increase of PWPU content to an intermediate level of 10%, as illustrated in Figure 9b, the failure pattern significantly evolves. A visible barreling effect already begins to emerge at this stage, indicating a transition towards ductile deformation. Although traces of a diagonal shear band remain discernible, the strain concentration is considerably alleviated, and the deformation exhibits a hybrid characteristic combining localized shearing and global radial expansion. This suggests that the polymer network is sufficiently developed to distribute stresses more effectively, initiating the shift from brittle fracture to ductile flow. In contrast, increasing the PWPU content to 15% (Figure 10b) significantly enhances the structural integrity. The high-strain zone becomes markedly diffuse and covers a larger surface area, manifesting as a barreling effect. Consequently, the peak stress increases to approximately 3.0 MPa, and the strain capacity improves remarkably. This comparison indicates that a higher density of polymer cross-links creates a robust 3D network that effectively bridges soil particles, redistributing local stress concentrations and suppressing the premature formation of shear bands [26].

The DIC observations align perfectly with the AE characteristics. The temporal release of AE energy aligns perfectly with the spatial evolution of strain fields. For the brittle specimens (e.g., *w* = 5%, PU = 10%), the quiet period in AE ringing counts corresponds to the uniform strain distribution observed in the DIC elastic stage (Point A). Crucially, the explosive surge in AE counts near the peak stress coincides exactly with the rapid nucleation of the high-intensity shear band in the DIC contour (Point B). The abrupt step-like jump in cumulative AE counts signifies the instantaneous release of strain energy, which manifests macroscopically as the sharp discontinuity in the horizontal displacement field. This confirms that the catastrophic failure is driven by the synchronized coalescence of internal micro-fractures into a singular through-going shear plane. Conversely, for the ductile specimens (e.g., *w* = 15%, PU = 10%), the continuous and steady AE activity throughout the post-yield stage corroborates the diffuse barreling deformation pattern. The absence of a sudden AE energy burst correlates with the suppression of strain localization in the DIC images. The damage does not concentrate in a narrow zone but disperses across the entire specimen volume, allowing the material to dissipate energy progressively through the global deformation of the polymer network [36].

The fracture mode classification based on RA-AF parameters provides a physical explanation for the kinematic behavior observed by DIC. The statistical dominance of tensile micro-cracks identified in Figure 8 is the microscopic origin of the macroscopic lateral expansion observed in Figure 9c and Figure 10b. Although the specimen is subjected to macroscopic compression, the bridge effect of the PWPU network constrains the relative sliding of soil particles. Consequently, the external compressive load is converted into internal tensile stresses acting on the polymer ligaments. The extensive stretching and tearing of these ligaments generate the tensile AE signals, which macroscopically manifest as the uniform radial expansion rather than diagonal shearing. This multi-scale corroboration confirms that the high ductility of the reinforced soil stems from the tensile capacity of the polymer matrix governing the global deformation [37].

### 3.4. Microscopic Mechanism Analysis of Reinforced Soil

**Figure 11:**
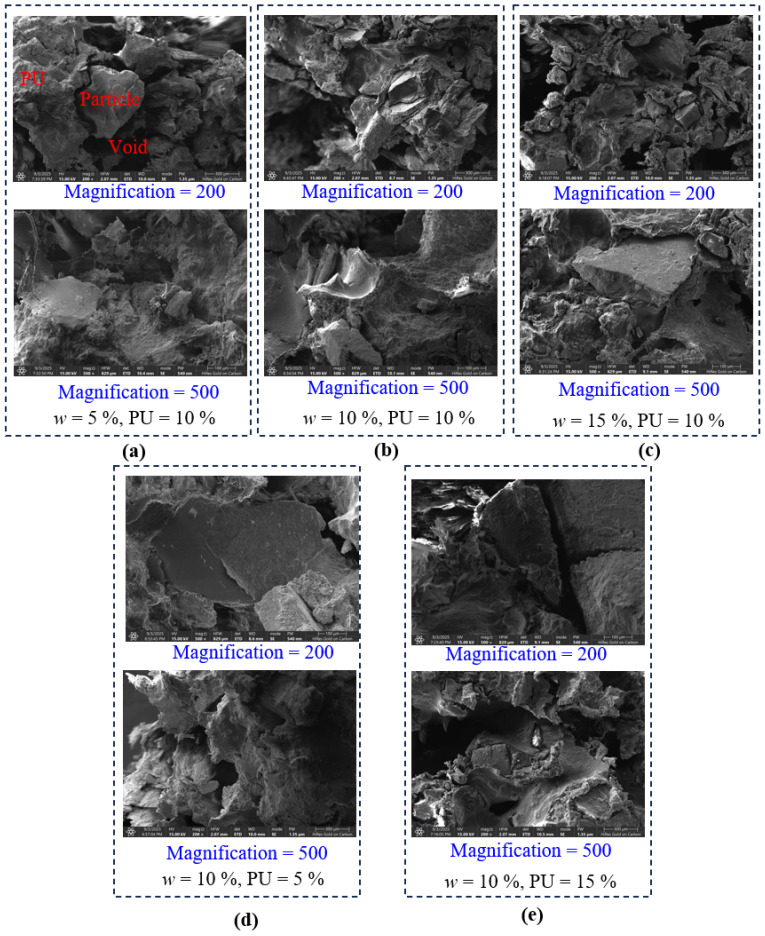
SEM micrographs showing the microstructural characteristics of PWPU-GRS under various initial water contents (*w*) and PWPU contents (PU) at magnifications of 200× and 500×: (**a**) *w* = 5%, PU = 10%; (**b**) *w* = 10%, PU = 10%; (**c**) *w* = 15%, PU = 10%; (**d**) *w* = 10%, PU = 5%; and (**e**) *w* = 10%, PU = 15%.

Figure 11 presents the SEM micrographs of the PWPU-GRS specimens under various initial water contents (*w*) and PU contents at magnifications of 200× and 500×. The micro-morphology reveals the interaction mechanisms between the polymer matrix and the soil particles, which can be categorized into surface wrapping, inter-particle bridging, and void filling.

Figure 11a–c illustrate the microstructural evolution as the initial water content increases from 5% to 15%. At the low water content of 5%, the reaction between the PWPU and the limited available water is constrained. Consequently, the polymer appears as discrete clusters or thin films adhering to the surface of the particles. The particle and void regions remain distinct, with numerous interconnected pores remaining open. The bonding mechanism here is primarily characterized by localized adhesion, which provides limited shear resistance. With increased water availability, the polymer exhibits a more expanded structure. The reaction between the PWPU prepolymer and water generates carbon dioxide, causing the PWPU to expand and form a reticular foam structure. This expansion facilitates better spatial distribution, allowing the binder to bridge adjacent particles more effectively [38]. At the highest water content of 15%, the expansion of the PWPU is most pronounced. The PU matrix covers a larger surface area of the aggregates, creating a continuous membrane that encapsulates the particles. However, excessive foaming driven by high water content may result in a more porous PWPU matrix itself, potentially altering the local stiffness of the bond [22].

Figure 11b,d,e demonstrate the effect of increasing PU content from 5% to 15%. At the low PU content of 5%, the quantity of binder is insufficient to form a continuous network. Most soil particles remain exposed, and the PWPU exists primarily as isolated spot welds at particle contact points. The large voids visible at 200× magnification indicate that the soil skeleton is not fully stabilized, suggesting lower macroscopic strength. In contrast, at the high PU content of 15%, a dense and cohesive network is observed. The voids are significantly reduced as they are filled by the cured PWPU matrix. The soil particles are deeply embedded within the PU, transitioning the material behavior from a granular soil governed by friction to a PWPU-matrix composite governed by the cohesion of the binder. The thick PWPU bridges visible at 500× magnification provide substantial resistance against particle rotation and displacement.

The SEM analysis confirms that the strengthening mechanism is twofold. The PU acts as a bonding agent that wraps around irregular particle surfaces and forms bridges between grains, enhancing mechanical interlock. Increasing both *w* and PU leads to a reduction in effective porosity. The expanded PWPU fills the inter-granular voids, restricting the movement of particles and densifying the soil fabric.

## 4. Conclusions

This study systematically investigated the mechanical performance and multi-scale failure mechanisms of PWPU-GRS. Based on the results from uniaxial compression tests, synchronous AE and DIC monitoring, and SEM analysis, the following conclusions can be drawn:

(1) The addition of PWPU significantly transforms the mechanical response of GRS from brittle failure to ductile yielding. The failure mode is governed by the coupling effect of initial water content (*w*) and PWPU content (PU). An optimal PU content of 10% was identified to maximize the compressive strength. Crucially, increasing the water content from 5% to 15% induces a shift from strain-softening to strain-hardening, with failure strains increasing linearly with PWPU dosage.
(2) Synchronous monitoring revealed distinct damage accumulation patterns. Brittle failure observed under low water and PWPU contents is characterized by localized shear bands and burst-type AE signals with step-like energy release. Conversely, ductile failure associated with high water and PWPU contents exhibits diffuse barreling deformation accompanied by continuous, low-amplitude AE activity. RA-AF analysis confirms that the failure mechanism shifts from shear-dominated friction to tensile-dominated fracturing involving the stretching of PWPU ligaments as the material becomes more ductile.
(3) DIC analysis visually quantified the capacity of the PWPU network to suppress strain localization. In brittle specimens, failure is driven by the rapid nucleation of a singular, high-intensity diagonal shear band. However, under optimal reinforcement conditions (PU = 10%, *w* = 10%), the polymer network effectively redistributes stress concentrations, preventing the premature formation of shear planes. Consequently, the deformation mode transforms into a diffuse barreling pattern, allowing the composite to sustain high axial loads even at large deformations.
(4) SEM observations elucidated the physical basis of the macroscopic enhancement. The water-reactive foaming process creates a porous, reticulated polymer structure that stabilizes the soil through three primary mechanisms: the wrapping effect, the bridging effect, and the void-filling effect. The cellular foam formed at higher water contents acts as a micro-cushion, where the flexible pore walls undergo bending and stretching. This structure provides the necessary flexibility for strain-hardening behavior while preserving the hydraulic permeability of the soil.

In summary, this study demonstrates that PWPU offers a promising solution for stabilizing granite residual soil by resolving the critical conflict between mechanical reinforcement and groundwater drainage. The established failure mechanisms, characterized by the transition from brittle fracture to ductile yielding, provide a theoretical foundation for designing resilient slopes that can withstand deformation without catastrophic collapse. Building on these mechanical insights, future research will focus on the quantitative assessment of hydraulic conductivity under varying stress states and the evaluation of the long-term durability of the polymer-soil composite in complex field environments, thereby advancing the practical engineering application of this technique.

## Figures and Tables

**Table 1:** Chemical component of the test soil measured by X-ray Fluorescence Spectrometer (XRF).

Component	Na_2_O	MgO	Al_2_O_3_	SiO_2_	K_2_O	Fe_2_O_3_	Others
Percent (%)	3.36	1	24.96	59	5	3.73	2.95

## Data Availability

Data will be made available on request.

## Abbreviations

The following abbreviations are used in this manuscript:

AE	Acoustic emission
AF	Average Frequency
DIC	Digital image correlation
GRS	Granite residual soil
PU	Polyurethane
PWPU	Permeable water-reactive polyurethane
PWPU-GRS	PWPU-treated GRS
RA	Rise Time/Amplitude
SEM	Scanning electron microscopy
UCS	Uniaxial compressive strength
XRD	X-ray diffraction
XRF	X-ray Fluorescence Spectrometer
